# Side-scan sonar imaging data of underwater vehicles for mine detection

**DOI:** 10.1016/j.dib.2024.110132

**Published:** 2024-02-01

**Authors:** Nuno Pessanha Santos, Ricardo Moura, Gonçalo Sampaio Torgal, Victor Lobo, Miguel de Castro Neto

**Affiliations:** a Portuguese Military Research Center (CINAMIL), Portuguese Military Academy (Academia Militar), Lisbon 1169-203, Portugal; b Institute for Systems and Robotics (ISR), Instituto Superior Técnico (IST), Lisbon 1049-001, Portugal; c Portuguese Navy Research Center (CINAV), Portuguese Naval Academy (Escola Naval), Almada 2810-001, Portugal; d Centro de Matemática e Aplicações (Nova Math), Universidade Nova de Lisboa, Caparica 2829-516, Portugal; e NOVA Information Management School (Nova IMS), Universidade Nova de Lisboa, Lisbon 1070-312, Portugal

**Keywords:** Autonomous underwater vehicles, Unmanned underwater vehicles, Sonar measurements, Sonar detection, Side-scan sonar

## Abstract

Unmanned vehicles have become increasingly popular in the underwater domain in the last decade, as they provide better operation reliability by minimizing human involvement in most tasks. Perception of the environment is crucial for safety and other tasks, such as guidance and trajectory control, mainly when operating underwater. Mine detection is one of the riskiest operations since it involves systems that can easily damage vehicles and endanger human lives if manned. Automating mine detection from side-scan sonar images enhances safety while reducing false negatives. The collected dataset contains 1170 real sonar images taken between 2010 and 2021 using a Teledyne Marine *Gavia* Autonomous Underwater Vehicle (AUV), which includes enough information to classify its content objects as NOn-Mine-like BOttom Objects (NOMBO) and MIne-Like COntacts (MILCO). The dataset is annotated and can be quickly deployed for object detection, classification, or image segmentation tasks. Collecting a dataset of this type requires a significant amount of time and cost, which increases its rarity and relevance to research and industrial development.

Specifications Table

**Table:** 

Subject	Computer Vision and Pattern Recognition
Specific subject area	Side-scan sonar imaging for NOn-Mine-like BOttom Objects (NOMBO) and MIne-Like COntacts (MILCO) object detection.
Data format	Raw and Processed data.
Type of data	.jpg and .txt files (dataset with the captured sonar images and the respective annotation).
Data collection	The data was collected *in situ* using a 900–1800 kHz Marine Sonic dual frequency side-scan sonar of a Teledyne Marine *Gavia* Autonomous Underwater Vehicle (AUV) between 2010 and 2021. Then, all the data was carefully analyzed, and each image was annotated with the NOn-Mine-like BOttom Objects (NOMBO) and MIne-Like COntacts (MILCO) object type Bounding Box (BB) coordinates. • Year: 2010 | Images: 345 | MILCO occurrences: 22 | NOMBO occurrences: 12 • Year: 2015 | Images: 120 | MILCO occurrences: 238 | NOMBO occurrences: 175 • Year: 2017 | Images: 93 | MILCO occurrences: 28 | NOMBO occurrences: 2 • Year: 2018 | Images: 564 | MILCO occurrences: 95 | NOMBO occurrences: 46 • Year: 2021 | Images: 48 | MILCO occurrences: 49 | NOMBO occurrences: 0
Data source location	The data was collected over the years along the Portuguese coast during missions performed by the Portuguese Navy's sappers’ divers group number three (*Destacamento de Mergulhadores Sapadores* - DMS 3). This group is responsible for all activities related to mine warfare at sea.
Data accessibility	Repository name: *Figshare* All data can be accessed at the following link: https://dx.doi.org/10.6084/m9.figshare.24574879 The archive content can be publicly accessed and downloaded without needing any registration.

## 1 Value of the Data

• Using this data can unlock the full potential of sonar image object detection and classification, facilitating the development of new algorithms and applications.
• These data were gathered in real-time using a state-of-the-art Autonomous Underwater Vehicle (AUV) and hold immense potential for validating developed models. Utilizing this data makes it possible to unlock new research possibilities, enabling the achievement of breakthroughs that would otherwise be impossible.
• Utilizing this dataset allows the researcher to eliminate the costly and time-intensive process of collecting field data. This will save resources and enable the researchers to focus on the critical aspects of their projects.
• Investing in a real dataset is a crucial step towards improving the safety and efficacy of mine detection systems. By incorporating real-world data, we can significantly reduce the probability of accidents and enhance the existing architectures to serve their purpose better.
• Civilian and military scientists can benefit from accurate data gathered by AUVs, which have already been annotated, facilitating algorithms and machine learning implementations.

## 2 Background

The data was collected over the years along the Portuguese coast during missions performed by the Portuguese Navy's sappers' divers group number three (*Destacamento de Mergulhadores Sapadores* - DMS 3). Due to the research and development efforts developed by the Portuguese Navy (PoN), every opportunity is used to gather data that is usually used when participating in national and international projects in the maritime domain. The last project where the PoN Research Center (*Centro de Investigação Naval* - CINAV) was responsible for the gathering and use of underwater sonar images for mine detection was the Open Cooperation for European Maritime Awareness (OCEAN 2020) project, funded under the European Union's Preparatory Action Plan on Defence Research (PADR) that occurred between 2018 and 2021 [1]. This project supported maritime surveillance and interdiction missions at sea using Unmanned Vehicles (UVs) integrated into fleet operations [2].

## 3 Data Description

**Fig. 1:**
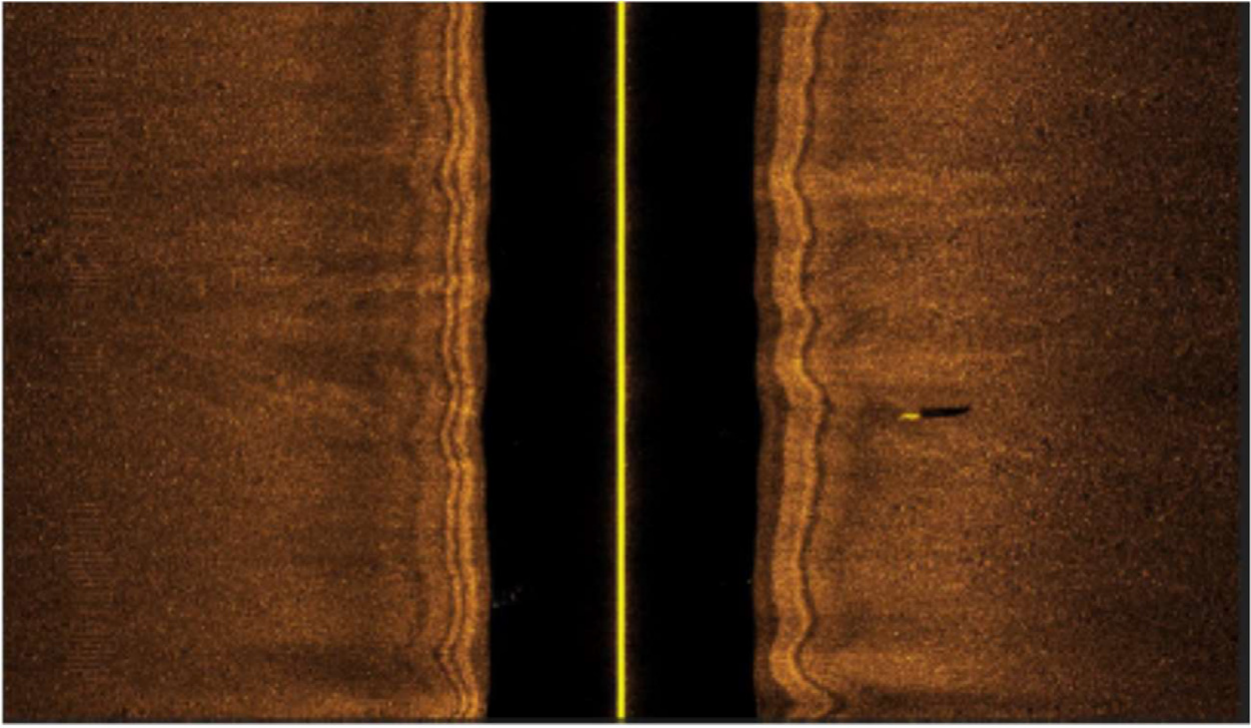
Example of a side-scan sonar image.

The dataset contains 1170 side-scan sonar images [3] collected using a 900–1800 kHz Marine Sonic dual frequency side-scan sonar of a Teledyne Marine *Gavia* Autonomous Underwater Vehicle (AUV) [4], as illustrated in Fig. 1. All the images were carefully analyzed and annotated, including the image coordinates of the Bounding Box (BB) of the detected objects divided into NOn-Mine-like BOttom Objects (NOMBO) and MIne-Like COntacts (MILCO) classes. The number of dataset images and the respective number of MILCO and NOMBO occurrences per year are described in Table 1.

**Table 1:** Summary of the dataset.

Date	Images	MILCO	NOMBO
2010	345	22	12
2015	120	238	175
2017	93	28	2
2018	564	95	46
2021	48	49	0

**Fig. 2:**
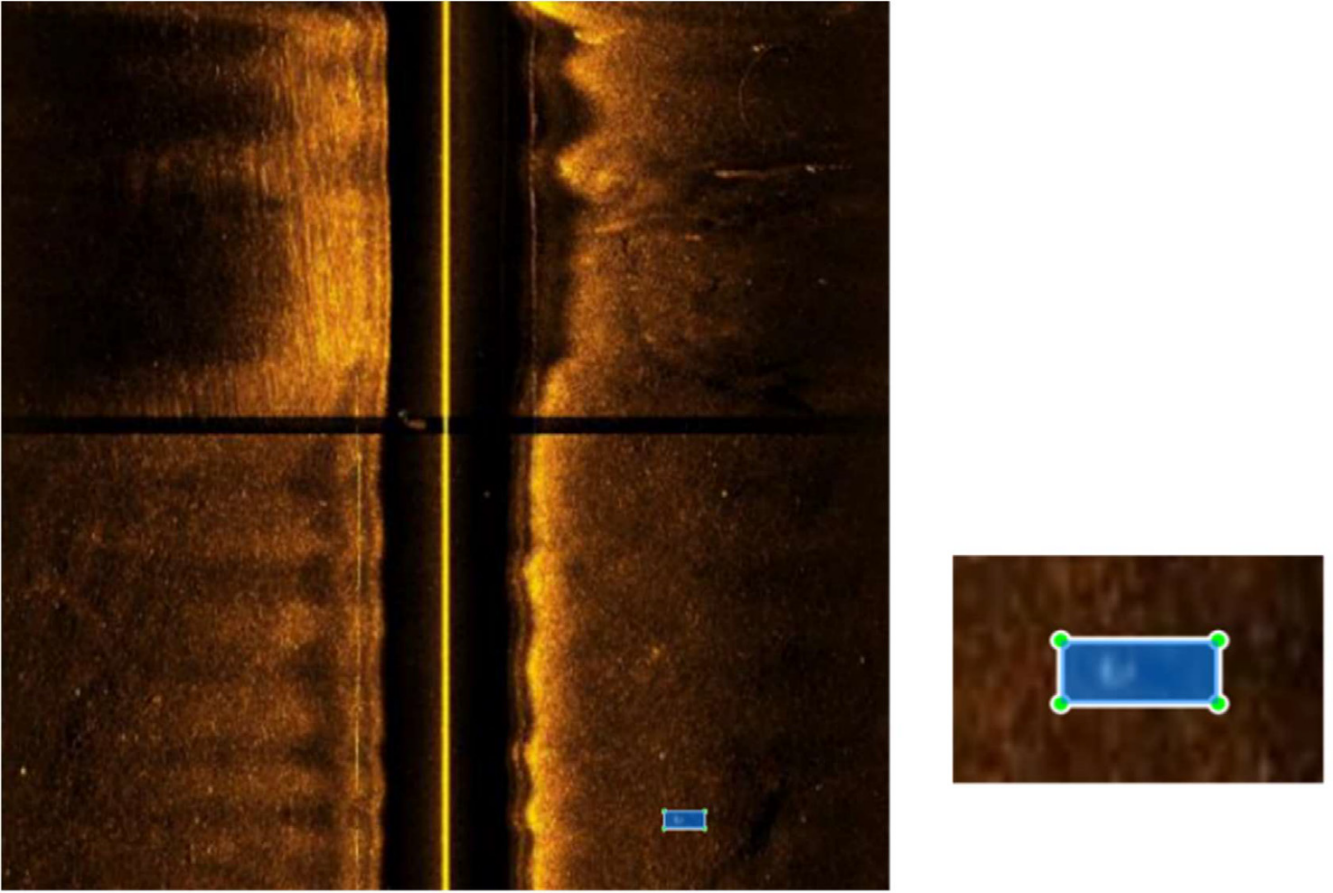
Side-scan sonar image showing the location of a MILCO object, represented by the BB given by $\left(x_{BB},y_{BB},w_{BB},h_{BB}\right)=\left(0.768,\;0.918,\;0.047,\;0.021\right)$.

**Fig. 3:**
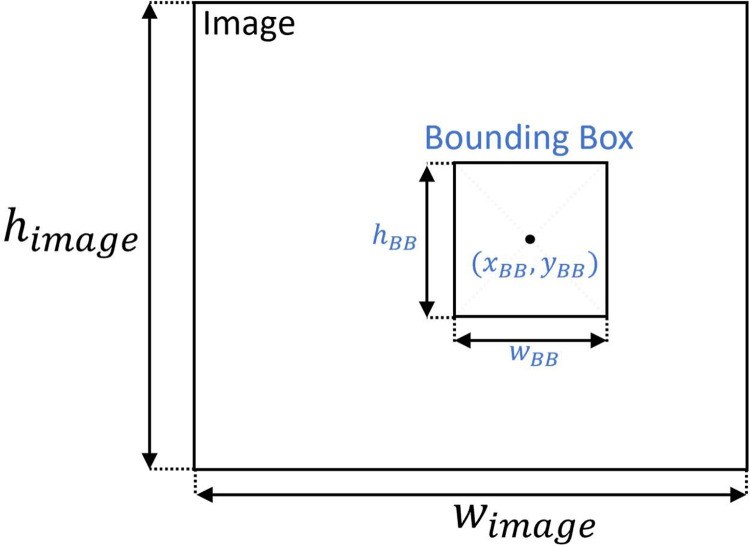
Image annotation illustration following the YOLO format.

**Fig. 4:**
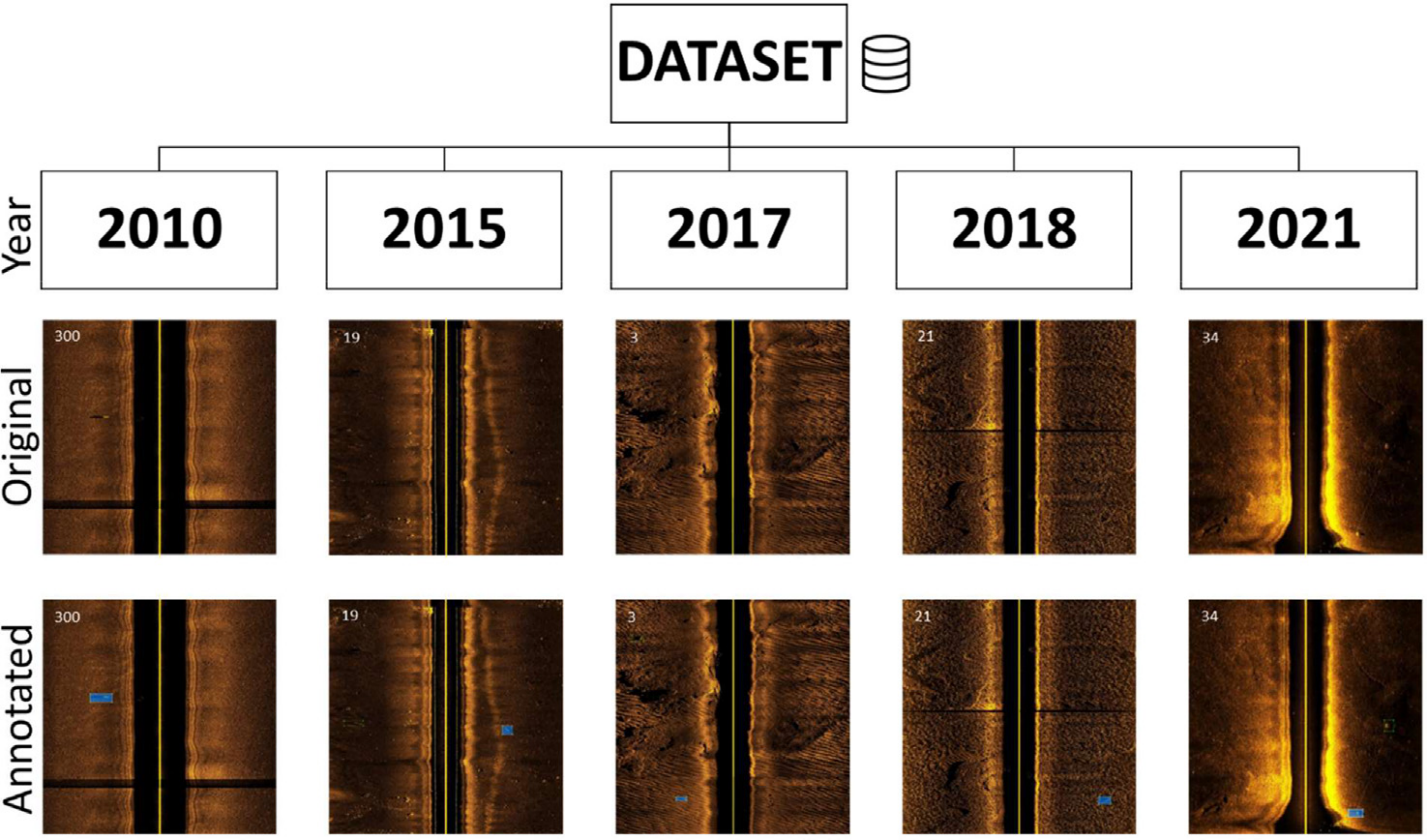
Examples of annotated images per year, with the image number in the top left corner.

The dataset is divided into five “.zip” files, one for each considered year, containing the numbered images “*XXXX_YYYY*.jpg” and an annotation file per image “*XXXX_YYYY*.txt” with the same number. The description *“XXXX”* corresponds to the index for a specific year, and “*YYYY*” corresponds to the considered year. The annotations include the object class and the BB coordinates of the object in the image. The annotation format is described in Table 2, where one example was provided, as illustrated in Fig. 2. The class “0” corresponds to a MILCO object, and the class “1” corresponds to a NOMBO object. The BB follow the representation illustrated in Fig. 3, and the absolute object BB coordinates (x,y,w,h) can be obtained using the following relation:

(1) $$\begin{array}{c} \left(x_{\mathrm{BB}},y_{\mathrm{BB}},w_{\mathrm{BB}},h_{\mathrm{BB}}\right)=\left(\frac{x}{w_{\mathrm{image}}},\frac{y}{h_{\mathrm{image}}},\frac{w}{w_{\mathrm{image}}},\frac{h}{h_{\mathrm{image}}}\right) \end{array}$$

where x,y are the absolute coordinates of the BB in the image and w,h are its absolute width and height. This representation follows the *standard* You Only Look Once (YOLO) object detection deep neural network format [5], and the annotation can be easily verified using *LabelImg,* a graphical image annotation tool commonly used during the image annotation process [6]. It is also possible to use *LabelImg* to convert the performed annotations to Pattern Analysis, Statistical Modelling, and Computational Learning (PASCAL) Visual Object Classes (VOC) [7], the format used by ImageNet [8] or Create Machine Learning (CreateML) [9] formats. Fig. 4 illustrates annotated images from each year, showing the complexity of the annotation process.

**Table 2:** Annotation format description with an example.

Class	Center coordinate $x_{BB}$	Center coordinate $y_{BB}$	Height $h_{BB}$	Width $w_{BB}$
0	0.768	0.918	0.047	0.021

**Fig. 5:**
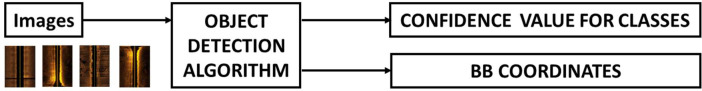
– *Standard* schematic used for object detection in CV.

Object detection is a common task in Computer Vision (CV), which involves estimating the coordinates of the bounding box of objects in an image. Regardless of the object detection algorithm used, the annotation format must be adapted or converted to suit the chosen architecture. The dataset provides annotations in the YOLO format, but using *LabelImg* or another equivalent tool, it can be easily converted as required. Fig. 5 shows a standard schematic used for object detection, where most algorithms, after being trained (in supervised training), provide confidence values for the classes and the respective estimated BB coordinates.

To perform an initial object detection using the provided dataset, we have implemented the YOLO v4 algorithm [10]. The necessary files were customized to meet the specific requirements of our object detection task, changing the original “*yolov4.cfg*” file to configure the batch size (number of samples used in a single training iteration) to 64, the subdivisions (assumed division in the training batches) to 16, the maximum number of batches (after reaching this value the training stops) to 6000, and configuring steps (batches were the learning rate is adjusted multiplying it by a 0.1 factor) at 4800 and 5400. We also set the network image input dimensions to 512 × 512 pixels (width x height) to optimize detection accuracy. As initial implementation, we have initialized the network weights with pre-trained values in the Common Objects in Context (COCO) dataset [11] using the file “*'yolo4.conv.137*” to perform transfer learning. In this preliminary test, using our dataset of 1170 images, over the first 5000 training iterations, we achieved an average Intersection over Union (IoU) of 60%, a mean Average Precision (AP) of 75%, a Precision of 82%, and a Recall of 64% with a confidence threshold of 0.25. All the considered files, including the final training weights “*yolov4-custom_5000.weights*” and a *Jupyter Notebook* example “*Real_time_object_classifier.ipynb*” with some implementation notes, are available in the dataset repository in a separate file named “Training.zip.” It is worth noting that this implementation provides a basic algorithm for initial object detection training. With detailed tuning and optimization of the model parameters and training process, significantly better results can be achieved.

## 4 Experimental Design, Materials and Methods

**Fig. 6:**
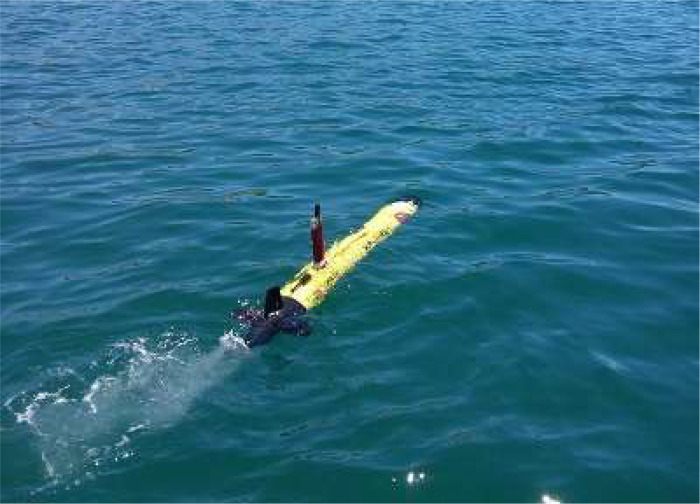
– Teledyne Marine Autonomous Underwater Vehicle (AUV).

As described, the dataset was acquired using a 900–1800 kHz Marine Sonic dual frequency side-scan sonar of a Teledyne Marine *Gavia* Autonomous Underwater Vehicle (AUV) [4], as illustrated in Fig. 6. The vehicle is composed of several modules that are 200 mm in diameter. These modules can be adapted to fit a variety of AUV missions. The vehicle's weight can range from 48 to 100 kg, and its size can vary from 1.7 to 3 m. The maximum depth that the vehicle can reach is 200 m, and its maximum speed is 5 knots. In addition to the side-scan sonar described earlier, the vehicle is also equipped with a Global Positioning System (GPS), an inertial navigation system, and a high-resolution camera.

## Limitations

In the world of data, particularly in image processing, the more images there are, the better it is to apply and develop algorithms. The dataset presents 1170 side-scan sonar images, which can be limited for some application and algorithm development.

## Ethics Statement

The authors confirm that the current work does not involve human subjects, animal experiments, or data collected from social media platforms, complying with all ethical requirements for publication in Data in Brief.

## CRediT authorship contribution statement

**Nuno Pessanha Santos:** Conceptualization, Validation, Formal analysis, Writing – original draft, Writing – review & editing, Visualization, Supervision, Project administration, Funding acquisition. **Ricardo Moura:** Conceptualization, Methodology, Validation, Formal analysis, Data curation, Writing – review & editing, Visualization, Supervision, Project administration, Funding acquisition. **Gonçalo Sampaio Torgal:** Methodology, Software, Validation, Formal analysis, Resources, Data curation, Visualization. **Victor Lobo:** Conceptualization, Project administration, Funding acquisition. **Miguel de Castro Neto:** Project administration, Funding acquisition.

## Data Availability

Side-scan sonar imaging for Mine detection (Original data) (Figshare).

## References

[1] European Commission, “OCEAN 2020: The EU's largest collaborative defence research project under the PADR successfully completed,” 2021. [Online]. Available: https://defence-industry-space.ec.europa.eu/ocean-2020-eus-largest-collaborative-defence-research-project-under-padr-successfully-completed_en. (Accessed 6 January 2024).

[2] European Union, “Open Cooperation for European mAritime awareNess,” 2021. [Online]. Available: https://defence-industry-space.ec.europa.eu/document/download/f0316a47-1b9b-4a81-9424-18b6cbcda350_en?filename=PADR%202017%20-%20OCEAN2020.pdf. [Accessed 07 January 2023].

[3] N. Pessanha Santos, R. Moura, G. Sampaio Torgal, V. Lobo and M. de Castro Neto, “Side-scan sonar imaging for Mine detection,” 2023. [Online]. Available: 10.6084/m9.figshare.24574879. PMC10879765 38384311

[4] T. Marine, “Gavia A.U.V.,” 2023. [Online]. Available: https://www.teledynemarine.com/en-us/products/Pages/gavia-auv.aspx.

[5] Redmon J., Farhadi A. (2017). IEEE Conference on Computer Vision and Pattern Recognition.

[6] T. Lin, “LabelImg,” [Online]. Available: https://github.com/HumanSignal/labelImg. (Accessed 01 December 2023).

[7] Everingham M., Ali Eslami S.M., Van Gool L., Williams C.K.I., Winn J., Zisserman A. (2015). The PASCAL visual object classes challenge: a retrospective. Int. J. Comput. Vis..

[8] Stanford Vision and Learning Laboratory (2020). https://www.image-net.org/index.php.

[9] Wang T., Zhu Y. (2022). International Conference on Cloud Computing, Performance Computing, and Deep Learning (CCPCDL 2022).

[10] A. Bochkovskiy, “darknet,” 2020. [Online]. Available: https://github.com/AlexeyAB/darknet. (Accessed 9 January 2024).

[11] Lin T.-Y., Maire M., Belongie S., Hays J., Perona P., Ramanan D., Dollár P., Zitnick C.L. (2014). European Conference on Computer Vision (ECCV).

